# Letter to the editor: Is malaria re-emerging in southern Europe? Cryptic *Plasmodium falciparum* malaria in Malta, October 2018

**DOI:** 10.2807/1560-7917.ES.2018.23.50.1800650

**Published:** 2018-12-13

**Authors:** Raquel Medialdea-Carrera, Tanya Melillo, Charmaine Gauci, Graziella Rocco, Maria Louise Borg

**Affiliations:** 1 Infectious Disease Control Unit; Health Promotion and Disease Prevention Directorate, Msida, Malta; 2 European Programme for Intervention Epidemiology Training (EPIET), European Centre for Disease Prevention and Control (ECDC), Stockholm, Sweden

**Keywords:** malaria, Plasmodium falciparum, Anopheles, Malta, vector control, infection control, Italy, outbreaks, parasitic infections, Plasmodium, surveillance, vector-borne infections

**To the editor:** We read with great interest the rapid communication by Zammarchi et al. [1] describing a cryptic severe *Plasmodium falciparum* malaria infection in a Moroccan man living in Tuscany, Italy. The authors described that the case was unlikely to be travel-associated or a malaria relapse. Moreover, they ruled out other possible risk factors, such as transfusion, transplant or nosocomial exposure. Thus, the authors reported the occurrence of a sporadic autochthonous *P. falciparum* infection in Italy, almost 50 years after the elimination of malaria in southern Europe in the 1970s.

In light of this unexpected case, we wish to report a cryptic case of *P. falciparum* diagnosed in October 2018 in Malta. A previously healthy man in his early thirties was admitted to Mater Dei Hospital in mid-October 2018 with an 8-day history of high fever, headache, vomiting, loss of appetite and dark urine. Blood tests showed anaemia (13.0g/dL; norm: 14.1–17.2g/dL), thrombocytopenia (101×10^9^/L; norm: 146–302×10^9^/L), low eosinophils (0.00×10^9^/L; norm: 0.10–0.70×10^9^/L), low lymphocyte count (0.92×10^9^/L; norm: 1.30–3.60×10^9^/L) and increased serum CRP (100.7mg/L norm; 0–5mg/L).

Examination of blood smears showed the presence of *P. falciparum* in peripheral blood and PCR results confirmed *P.* *falciparum* infection with high viraemia. The patient was treated with three doses of atovaquone/proguanil (1g/400mg per day) and was discharged 4 days after hospital admission. The patient made a full recovery within 7 days.

The epidemiological investigation revealed that the patient, a resident of central Malta, was born in Burkina Faso and has been living in Malta for over 10 years. The patient reported having had malaria in 2007, when they were still living in their home country. Later that year, the patient arrived as a migrant to Malta. They had not travel abroad since then. The patient works discontinuously, mainly outdoors, and reported being unemployed prior to symptom onset. The patient has no history of surgery, blood transfusion or invasive examination, denied being an injecting drug user and was not aware of having had contact with persons with similar symptoms. The patient’s residence is located ca 7 km from Malta International Airport. They noticed multiple mosquito bites during the weeks preceding illness. Extensive investigations failed to identify the potential source of this sporadic case and there was no evidence of further local transmission.

During 2018, another four cases of malaria were reported in Malta (three *P. falciparum* and one *P. vivax*). All of them were imported cases associated with recent travel to Asia or Africa. No cases of locally acquired malaria have been reported in Malta since the early 1940s, and *Anopheles* spp. have not been detected since its eradication in 1943 [2]. The most recent entomological investigations conducted in 2010 and 2013, involving both larval collection and adult trapping, reconfirmed the presence of *Culex pipiens* and demonstrated that *Aedes albopictus* has been introduced and established in the Maltese Islands [3]. However, no *Anopheles* spp. were identified in these investigations.

Although unlikely, a plausible explanation may be that the patient had asymptomatic *P. falciparum* infection that persisted for over a decade. Recently, a case identified 13 years after initial exposure to *P. falciparum* malaria was reported [4].

Even though Malta is a small country, the high number of visiting tourists, along with increased numbers of migrants and favourable Mediterranean climate, increase the risk of sporadic autochthonous cases and establishment of malaria in the region. There exists an urgent need, both in Malta and in other European countries, for increased awareness of imported malaria as well as potential autochthonous malaria cases; further efforts should be made to heighten awareness among clinicians to ensure prompt detection, notification and treatment of cases, as well as timely implementation of public health control measures.

The unexpected finding of this cryptic *P. falciparum* malaria case in Malta suggests that competent vectors for transmission may be present. This event, together with the recent *P. falciparum* malaria case in Italy, indicates that re-establishment of malaria in southern Europe may be possible and reinforces the need for improved surveillance and urgent vector-control actions.
