# Authors’ correction for Euro Surveill. 2018;23(50)

**DOI:** 10.2807/1560-7917.ES.2018.24.2.190110c3

**Published:** 2019-01-10

**Authors:** Eurosurveillance editorial team

**Affiliations:** 1 European Centre for Disease Prevention and Control (ECDC), Stockholm, Sweden

On request of the authors, viraemia was corrected to parasitaemia in the Letter to the editor ‘Is malaria re-emerging in southern Europe? Cryptic Plasmodium falciparum malaria in Malta, October 2018’. This change was made on 10 January 2019. 

